# Driving factors of agricultural artificial intelligence adoption intention: an empirical study in Shandong province based on innovation characteristics, technology commitment, and individual heterogeneity

**DOI:** 10.3389/frai.2026.1630717

**Published:** 2026-02-19

**Authors:** Kai Cao, Ping Wang, Siyu Kong, Chunzhen Zhang

**Affiliations:** 1 Library of Qinghai University, Qinghai University, Xining, Qinghai, China; 2 School of International Education, Qinghai Minzu University, Xining, Qinghai, China; 3 Institute of Urban Agriculture, Chinese Academy of Agricultural Sciences, Chengdu, Sichuan, China

**Keywords:** adoption intention, agricultural artificial intelligence, individual heterogeneity, moderation effect, technology characteristics

## Abstract

In the “Agriculture 4.0 era,” the implementation of agricultural artificial intelligence (AI) has been proven to bring economic and environmental benefits to farmers. Despite its potential advantages, the adoption rate of agricultural AI remains relatively low. To explore the adoption driving mechanism of agricultural AI in major producing areas, this study took 359 agricultural practitioners in Shandong Province as samples, constructed an extended technology acceptance model (TAM)–unified theory of acceptance and use of technology (UTAUT) model integrating technological innovation characteristics, technology commitment, and individual heterogeneity, and used the partial least squares-structural equation modeling (PLS-SEM) method to empirically analyze the influencing factors and moderating effects of adoption intention. The results show that mobility, autonomy, technological interest, and technological control belief significantly and positively affect perceived ease of use; mobility, technological interest, and perceived ease of use have significant positive effects on perceived usefulness; perceived ease of use and perceived usefulness jointly drive the improvement of adoption intention. Educational background and work experience have significant moderating roles: Higher education strengthens the positive impact of technological interest on perceived ease of use, and rich work experience amplifies the promoting effect of technological competence belief (TCM) on perceived ease of use. However, the impacts of autonomy on perceived usefulness and technological competence belief on perceived ease of use and perceived usefulness are not statistically significant, which is closely related to the production characteristics of smallholder farmers and insufficient technological adaptability. This study improves the theoretical framework of agricultural AI adoption, provides an empirical basis for formulating differentiated technology promotion strategies and optimizing technology design, and has important practical significance for accelerating agricultural digital transformation.

## 1 Introduction

As the cornerstone of the national economy, the sustainable development of agriculture highly relies on the innovation and application of advanced agricultural technologies (Ableeva et al., 2019). Against this backdrop, modern smart agriculture driven by artificial intelligence (AI) has become a strategic priority in global agricultural development, attracting great attention and active promotion from all sectors of Chinese society. At the policy level, the 2023 Central No. 1 Document clearly proposes “promoting the R&D and application of smart agriculture” (Brief, 2023), and the state has invested over 5 billion yuan in special funds to support the R&D and implementation of agricultural AI projects during the “14th Five-Year Plan” period. The “Agricultural and Rural Big Data Platform” led by the Ministry of Agriculture and Rural Affairs has integrated more than 4 billion agricultural-related data entries, providing precise agricultural production predictions and decision support services for more than half of the counties nationwide (Jingwei, 2025). At the local level, Shandong Province, a major agricultural province, has also outlined the construction of a regionally distinctive “smart agriculture plan” in its “14th Five-Year Plan for Digital Shandong Construction,” committing to promoting the in-depth integration and innovative application of new-generation information technologies such as AI and the Internet of Things throughout the entire agricultural industrial chain.

In terms of industry–scientific research collaborative innovation, China Agricultural University and Tencent jointly established a Smart Agriculture Laboratory and successfully developed a high-precision crop growth simulation system (Wang, 2024). e-commerce giant Pinduoduo launched the “Agricultural AI Ecosystem Plan,” planning to invest 1 billion yuan in the R&D and ecological construction of agricultural AI technologies (Qiu et al., 2021). Within Shandong Province, institutions of higher learning such as Shandong Agricultural University and Qingdao Agricultural University have also actively carried out in-depth cooperation with local leading technology enterprises, including Haier and Inspur, to jointly tackle key agricultural AI technologies (Holzinger et al., 2022). This series of top–down policy guidance, capital investment, and industry–university–research collaborative innovation initiatives are systematically promoting the transformation and upgrading of China's traditional agriculture toward intelligence, digitalization, and precision.

The “International Assessment of Agricultural Knowledge, Science and Technology” (McDonald et al., 2016) has long pointed out that agricultural technological progress is the core key to addressing global food security, resource constraints, and environmental pressures, as well as achieving sustainable agricultural development. The academic community generally agrees that the extensive development of smart agriculture and the realization of sustainable agricultural goals are inseparable from the in-depth empowerment and widespread penetration of agricultural AI (Alzubi and Galyna, 2023; Gikunda, 2024; Singh and Jain, 2022; Ziesche et al., 2023). However, the core challenge of smart agriculture is not merely limited to the R&D and supply of agricultural AI technologies, but more importantly, to ensure that farmers, as the ultimate users of technologies, can truly understand, effectively accept, and actively adopt these innovative technologies (Yadav and Pathak, 2016). The vitality of new technologies lies in their application, and the breadth and depth of their promotion ultimately depend on users' acceptance intentions and adoption behaviors.

In the field of academic research, technology adoption models have become the core theoretical framework and empirical basis for exploring agricultural AI adoption intentions (Dissanayake et al., 2022; Dong et al., 2022; Mohr and Kühl, 2021; Sood et al., 2023). To effectively promote the adoption intention and in-depth application of agricultural AI technologies by farmers in Shandong Province and thereby accelerate the process of agricultural modernization, it is urgent to comprehensively and in-depth identify and analyze the key driving factors and potential obstacles affecting farmers' acceptance and adoption willingness of these new technologies. Although existing literature has extensively discussed agricultural AI adoption behaviors, there are still significant differences among different studies in terms of theoretical perspectives, model construction, and variable selection: Some studies focus on testing the applicability of the traditional technology acceptance model (TAM) (Mishra et al., 2024; Na et al., 2022) or the unified theory of acceptance and use of technology (UTAUT) (Abdollahzadeh et al., 2024; Nayal et al., 2022) in the agricultural AI context; other studies have explored supplementary factors beyond the explanatory power of existing variables by integrating or expanding classic models such as TAM and UTAUT (Eweoya et al., 2021); some studies have further explored the moderating effects of demographic characteristics or contextual factors on specific path relationships in the model.

Most of the above studies focus on agricultural AI adoption intention itself and mainly explore its influencing mechanisms from the perspectives of social influence factors (such as subjective norms and social networks) or individual psychological characteristics (such as perceived usefulness, perceived ease of use, and risk preference). However, this study aims to break through the limitations of existing research and construct a more comprehensive theoretical analysis framework based on the technology acceptance model (TAM) (Davis, 1989) and the unified theory of acceptance and use of technology (UTAUT) (Venkatesh et al., 2003).

This study will adopt a questionnaire survey method to collect primary data and use partial least squares structural equation modeling (PLS-SEM) for data analysis and model testing. On the basis of synthesizing previous research results, the innovations and expansion directions of this study are mainly reflected in two aspects: first, from the perspective of the attributes of technology itself, exploring the direct and indirect impacts of technological innovation (specifically focusing on the dimensions of autonomy and mobility) (Ghosh et al., 2024) and individuals' technology commitment levels (specifically including the dimensions of technological interest, technological competence belief (TCM), and technological control belief) (Rübcke von Veltheim et al., 2022) on farmers' AI technology adoption intentions; second, focusing on examining the potential moderating roles of key individual characteristics such as farmers' work experience and educational background in the above influence paths. Through this research design, it is expected to provide more precise and targeted practical implications and policy recommendations for the effective promotion and application of agricultural AI technologies in Shandong Province and even nationwide.

The selection of Shandong Province as the empirical object of this study is mainly based on its unique advantages and representativeness in agricultural development and agricultural AI application. First, Shandong Province is a well-deserved major agricultural province in China, with its comprehensive agricultural strength ranking among the top in the country for a long time, and it has significant first-mover advantages in the R&D and application of agricultural AI. For example, Shandong is a major national producer of grain, vegetables, fruits, and aquatic products, which provides a variety of application scenarios and broad market space for agricultural AI technologies (XiuYi, 2022). At the same time, the agricultural mechanization rate in Shandong Province has exceeded 88%, laying a solid hardware foundation for promoting AI technologies such as intelligent agricultural machinery and robot farms. Second, Shandong Province has unique geographical and climatic conditions, with complex and diverse landforms including plains, hills, and coasts, and climatic conditions suitable for the growth of various crops. This provides an ideal test bed for agricultural AI technologies to develop customized solutions for the personalized needs of different regions and crops, such as precision irrigation, intelligent pest and disease monitoring, and smart greenhouse management. For example, the developed marine fishery in the Jiaodong Peninsula provides unique scenarios for the application of AI in marine environment monitoring, intelligent aquaculture, and pelagic fishing (Wang J. et al., 2022). Third, the Shandong Provincial Government attaches great importance to and strongly supports the R&D and application of new-generation information technologies such as AI, the Internet of Things, and big data in the agricultural field, and has initially formed an industrial cluster effect of a certain scale (Wang Y. et al., 2022). For example, core cities such as Qingdao and Jinan have gathered leading domestic technology enterprises including Haier, Hisense, and Inspur, which can provide strong technical support and industrial guarantees for the development of agricultural AI equipment, data processing, and cloud platform services. Therefore, selecting Shandong Province as the research object has important theoretical and practical significance, and its research results will have strong representativeness and promotional value.

## 2 Literature review

Numerous landmark theories and models have been developed in the field of technology acceptance, adoption, and diffusion, such as the theory of reasoned action (TRA) (Fishbein and Ajzen, 1977), technology acceptance model (TAM) (Davis, 1985), theory of planned behavior (TPB) (Ajzen, 1991), diffusion of innovations theory (IDT) (Rogers et al., 2014), unified theory of acceptance and use of technology (UTAUT) (Venkatesh et al., 2003), social cognitive theory (SCT) (Bandura, 1986), and innovation characteristics theory (Hameed et al., 2012). Among them, the technology acceptance model (TAM) proposed by Davis serves as a foundational theoretical framework for technology usage research, aiming to predict users' behavioral attitudes and usage intentions toward new technologies. Its concise yet powerful predictive logic demonstrates significant applicability in the initial research phase of usage intentions for emerging technologies such as agricultural artificial intelligence (Bagozzi and Lee, 1999). Given that agricultural AI is still in the early stage of promotion and initial application, users' actual adoption behaviors may be constrained by various external environmental factors, including infrastructure, policy support, and cost investment, and often lag behind the formation of subjective intentions. Therefore, this study focuses on users' usage intentions rather than direct adoption behaviors to more accurately capture their initial attitudes and potential motivations toward agricultural AI technologies.

### 2.1 Technology acceptance model (TAM)

The technology acceptance model (TAM) was first proposed by Davis in 1985 based on the theory of reasoned action (TRA), aiming to explain and predict users' acceptance behaviors of information technologies. Its core constructs include user motivation variables—perceived usefulness and perceived ease of use—as well as outcome variables, namely behavioral intention and actual system use (Davis, 1985). Perceived ease of use and perceived usefulness are the key core variables in the TAM model that explain users' usage intentions and behaviors, together forming the psychological basis for users' acceptance of new technologies (Marangunić and Granić, 2015). Specifically, perceived ease of use refers to the level of effort users perceive when using a specific new technology, i.e., the perceived difficulty in learning and operating the technology; perceived usefulness reflects users' subjective belief that using a specific new technology can improve their work performance or bring specific benefits, especially the perceived advantages in terms of technical performance, such as improving production efficiency, reducing costs, or enhancing decision-making quality.

Since its proposal, the TAM model has been widely applied and validated in the field of information systems due to its simplicity and strong explanatory power, and it has spawned extended models such as TAM2 (Venkatesh and Davis, 2000) and TAM3 (Venkatesh and Bala, 2008). A large body of existing literature has verified the applicability and explanatory power of TAM and its extended models in the field of agricultural AI usage intentions. For example, a study by Caffaro et al. (2020) on the influencing factors of Italian farmers' adoption intentions toward smart farming technologies showed that a TAM-based mediation model indicated perceived usefulness as the core determinant driving farmers' AI adoption intentions, with its direct positive impact significantly higher than that of perceived ease of use. Similarly, when exploring the influencing factors of Chinese farmers' adoption intentions toward live-streaming e-commerce, Chen et al. (2024) found that external variables such as government support and social learning indirectly affect adoption intentions through the mediating role of perceived ease of use, while perceived usefulness directly and positively influences farmers' adoption decisions. These studies all demonstrate that the TAM model can effectively capture the psychological perception process of agricultural users toward emerging technologies and provide a solid theoretical foundation for understanding their usage intentions.

### 2.2 Unified theory of acceptance and use of technology (UTAUT)

The unified theory of acceptance and use of technology (UTAUT) was proposed by Venkatesh et al. (2003). This theory integrates the core findings and key constructs of eight mainstream theoretical models in previous technology acceptance research (including TRA, TAM, MM, IDT, SCT, etc.), aiming to more comprehensively and universally predict and explain the acceptance behaviors and usage of new technologies. A notable feature of UTAUT is its universality across different types of new technologies and good transferability across research contexts, enabling it to effectively address differences in technical characteristics and user groups. Therefore, its theoretical framework can provide a more comprehensive perspective for research on the adoption intentions of complex emerging technologies such as agricultural AI.

To better align with the specific context of agricultural AI adoption intentions in this study and improve the model's explanatory power and predictive accuracy, we targeted the adjustment and extension of the original core constructs of UTAUT (performance expectancy, effort expectancy, social influence, and facilitating conditions) by integrating the unique characteristics of agricultural AI and the behavioral traits of agricultural users. Specifically, the adjusted exogenous variables mainly include three core variables related to technology commitment (technological interest, technological competence belief, technological control belief) (Heerink et al., 2010; Kulviwat et al., 2007; Venkatesh et al., 2011) and two key variables related to technological innovation characteristics (mobility, autonomy) (Issa et al., 2022; Soma et al., 2019). These newly added constructs aim to more accurately capture users' intrinsic psychological tendencies toward agricultural AI and the unique attributes of the technologies themselves, thereby supplementing and improving the theoretical framework of the UTAUT model in explaining the adoption intentions of agricultural AI (Neyer et al., 2012).

Among the technology commitment variables, technological interest is a representative psychological attribute reflecting individuals' positive subjective evaluations and curious exploratory desires toward technological progress and its applications, embodying users' intrinsic preferences and willingness to actively approach technologies. Technological competence belief refers to users' subjective expectations and self-efficacy regarding their ability to effectively understand, learn, and apply a specific technology to solve practical problems and seize potential opportunities in a given technical context. As the third core dimension of technology commitment, technological control belief defines users' subjective expectations regarding the extent to which they can control the technology usage process, address potential problems during usage, and achieve expected outcomes, concentrating on users' perceived controllability of technology usage (Neyer et al., 2012).

Among the technological innovation characteristics variables, mobility endows agricultural AI equipment with the ability to move autonomously and perform human-like tasks in various complex farmland environments, such as field walking, water navigation, and aerial flight, which greatly expands the application scope and flexibility of the technology. Autonomy refers to the ability of agricultural AI systems or robots to independently perceive the environment, make decisions, and execute specific agricultural tasks without (or with minimal) human intervention, instructions, or continuous control (Bekey, 2012). The organic combination of these technological innovation characteristics and technology commitment variables can more comprehensively reflect the unique value of agricultural AI and users' subjective perceptions of them, thereby further improving the theoretical explanatory power of the UTAUT model for testing the impact of innovative technological characteristics on the acceptance and usage intentions of new technologies.

### 2.3 Theoretical model construction

**Figure 1:**
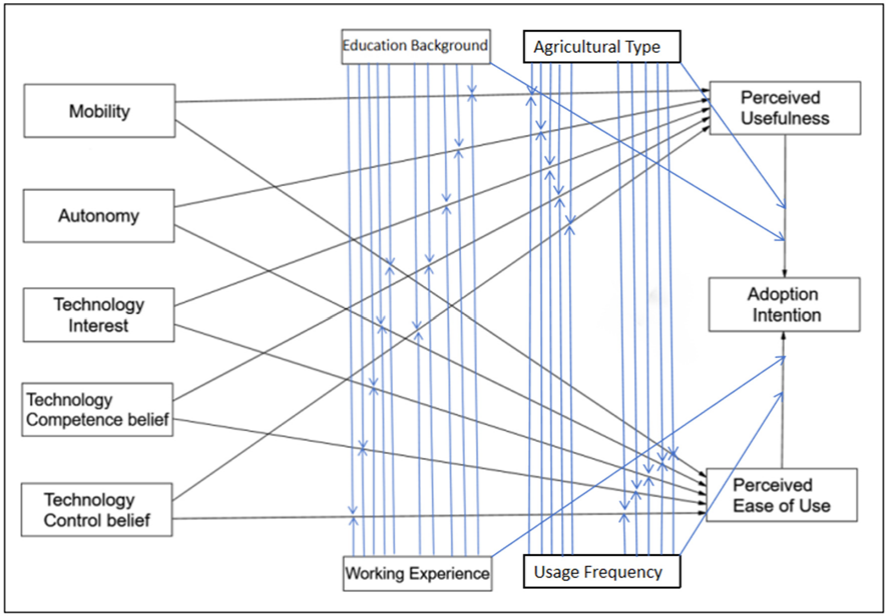
Theoretical model of agricultural AI adoption intention based on extended TAM-UTAUT. The model integrates technological innovation characteristics (mobility, autonomy), technology commitment (technological interest, technological competence belief, technological control belief), perceived variables (perceived ease of use, perceived usefulness), adoption intention, and individual heterogeneous moderating variables (educational background, work experience, agricultural type, usage frequency); TAM, technology acceptance model, UTAUT, unified theory of acceptance and use of technology.

Based on the aforementioned review and analysis of the technology acceptance model (TAM) and the unified theory of acceptance and use of technology (UTAUT), and taking full account of the characteristics of agricultural artificial intelligence technology and the actual circumstances of agricultural users, this study aims to construct an integrated theoretical framework to more systematically and in-depth examine the adoption intention of agricultural AI among agricultural users in Shandong Province. Specifically (as seen in Figure 1), this study plans to integrate the core mediating variables of perceived usefulness and perceived ease of use from the TAM, three core technology commitment-related variables (technology interest, technology competence belief, and technology control belief) derived from the extended UTAUT model, as well as two key technology innovation characteristic-related variables (mobility and autonomy) as the main exogenous variables of the research framework. Among these, perceived ease of use and perceived usefulness are designated as core mediating variables, responsible for transmitting the impacts of the aforementioned exogenous variables on the final outcome variable. Adoption intention, as the core outcome variable of this study, is used to measure the intensity of users' subjective intention to accept and use agricultural AI.

Furthermore, considering the heterogeneity of the agricultural user group, users with different demographic characteristics (such as educational background, farming styles, work experience, and usage frequency) may exhibit significant differences in their cognitive levels, learning abilities, risk preferences, and resource accessibility regarding new technologies. These differences may affect their evaluations of the perceived ease of use and perceived usefulness of agricultural AI, thereby moderating the intensity of the impacts of exogenous variables on mediating variables or the influence paths of mediating variables on the outcome variable. Therefore, this study further introduces demographic characteristics as potential moderating variables to test their moderating effects in the model, aiming to more elaborately reveal the formation mechanisms of adoption intention among different user groups.

Through the integration of the aforementioned variables and the setting of influence paths, this study ultimately constructs a more comprehensive and context-adaptive theoretical framework. It is expected to more comprehensively and in-depth analyze the complex influence mechanisms underlying the adoption intention of agricultural AI among agricultural users in Shandong Province, thereby providing a theoretical basis and practical implications for relevant policy formulation and technology promotion.

## 3 Development of research hypotheses

Based on the aforementioned theoretical framework, this study systematically proposes research hypotheses with the aim of rigorously examining the complex relationships among core variables and their ultimate impacts on farmers' intention to adopt agricultural AI. The derivation of all hypotheses is closely grounded in existing theoretical foundations and empirical findings from relevant literature, striving for the internal consistency of theoretical logic and the robustness of empirical support. The research hypotheses serve three key functions in this study: first, to clearly elaborate on the theoretical pathways and inherent mechanisms among technological innovation characteristics, technology commitment factors, perceptual variables, and adoption intention; second, to provide solid theoretical guidance and testable research propositions for subsequent empirical analysis; and third, to construct a structured research framework to effectively guide questionnaire design, data collection, statistical analysis, as well as the interpretation and discussion of research results.

### 3.1 Mobility

As a crucial enabling characteristic of agricultural AI, the core value of mobility lies in breaking the spatiotemporal constraints of traditional agricultural production. It allows farmers to access, learn, and apply AI anytime and anywhere through mobile terminals such as smartphones and wearable devices, thereby significantly reducing the physical barriers and initial obstacles to AI use (Elliott and Boyd, 2020). The inherent portability and contextual adaptability of mobile applications often imply more intuitive user interfaces and simplified operational processes, which directly facilitate the reduction of farmers' learning costs and operational complexity (Nah et al., 2005). This flexibility in technology access and convenience in operation can effectively enhance farmers' evaluations of perceived ease of use by improving the overall accessibility of agricultural AI. Meanwhile, mobility greatly expands the application boundaries of AI in agricultural scenarios, such as supporting remote expert collaborative diagnosis of plant diseases and pests and providing location-based real-time agricultural services (Paiva et al., 2021). These extended applications can strengthen farmers' recognition of the practical value of technical tools, making it easier for them to perceive the potential capabilities of AI in improving production efficiency, optimizing resource allocation, and meeting personalized needs (Ye et al., 2020), thereby enhancing their perceived usefulness of agricultural AI (Chinomona, 2013). Based on this, this study proposes the following hypotheses:

H1a: Mobility has a positive impact on perceived ease of use;

H1b: Mobility has a positive impact on perceived usefulness.

### 3.2 Autonomy

The concept of autonomy originally originated in the field of personality psychology to describe the degree of independence in an individual's thoughts and behaviors (Bublitz and Merkel, 2009). In recent years, it has been gradually extended to the fields of work tasks and organizational management and integrated into the theoretical frameworks of technological innovation and user behavior research (De Spiegelaere et al., 2016). As a key antecedent factor affecting job satisfaction, high performance, and service innovation capabilities (Demircioglu, 2021), autonomy in the application context of agricultural AI is mainly reflected in the independent decision-making and execution capabilities of technical systems, such as intelligent agricultural assistants and autonomous agricultural machinery. These autonomous technologies can significantly reduce users' manual operation steps and cognitive load by automating core operational processes (Beloev et al., 2021). For example, intelligent greenhouse temperature control systems can automatically adjust temperature and humidity according to preset algorithms and real-time environmental data, reducing the frequency and intensity of manual intervention by farmers, thereby enhancing their perceived ease of use of the technology; the autonomous inspection and precision spraying functions of agricultural drones improve operational accuracy and resource utilization efficiency by optimizing operational processes and reducing human operational errors (Moreno-Jacobo et al., 2021). This improvement in practical utility further enhances users' perceived usefulness of the technology (Yoon and Hwang, 2024). Therefore, this study hypothesizes:

H2a: Autonomy has a positive impact on perceived ease of use;

H2b: Autonomy has a positive impact on perceived usefulness.

### 3.3 Technological interest

As a key intrinsic motivational factor, technological interest profoundly drives individuals' willingness and behavioral tendencies to explore and adopt new technologies. When farmers hold a strong curiosity and exploratory desire toward agricultural AI itself, they are more likely to proactively invest time and energy in learning technical knowledge and overcoming potential learning barriers and usage anxiety (Hang, 2024). This intrinsic motivation triggered by interest can accelerate users' understanding and adaptation to the operational logic of technical interfaces and reduce their subjective perception of technical complexity, thereby enhancing the perceived ease of use of agricultural AI. Furthermore, farmers with strong technological interest often exhibit greater initiative in exploring and expanding the potential application value of technology: Technology enthusiasts may discover the efficiency improvement space or innovative application models of AI tools in specific agricultural scenarios earlier and actively integrate them into existing workflows, or even explore new application scenarios. This process of in-depth use and value excavation will significantly strengthen their perceived usefulness of the technology (Kim et al., 2021). Therefore, this study proposes

H3a: Technological interest has a positive impact on perceived ease of use;

H3b: Technological interest has a positive impact on perceived usefulness.

### 3.4 Technological competence belief

Technological competence belief includes users' subjective evaluation and confidence in their own digital skills, relevant prior knowledge, and ability to learn new technologies, which directly determines users' learning efficiency and usage effectiveness when facing new technologies. Generally speaking, users with higher technological competence belief, due to their existing knowledge reserves and learning abilities, can more quickly understand and master the operational processes and core functions of agricultural AI tools, thereby effectively reducing cognitive barriers and frustration during the learning process and enhancing the perceived ease of use of the technology (Ardiyanti and Susilowati, 2024a). Moreover, such users are more inclined to explore and utilize advanced functions of the technology or conduct personalized settings. This proactive exploration and in-depth use behavior, in turn, further optimizes their perceived ease of use of the technology (Adenekan et al., 2023). In addition, technological competence belief indirectly enhances users' perceived usefulness of agricultural AI in increasing yields and optimizing management by strengthening their sense of control and self-efficacy during technology use. Thus, this study hypothesizes

H4a: Technological competence belief has a positive impact on perceived ease of use;

H4b: Technological competence belief has a positive impact on perceived usefulness.

### 3.5 Technological control belief

Technological control belief specifically refers to farmers' subjective perception and confidence in their ability to effectively control the operational processes, outcomes, and inherent settings of agricultural AI during its use. When users perceive that they can exercise effective control over the technical system—such as customizing the operation interface according to personal habits, flexibly adjusting functional parameters, or modifying permission settings—their sense of uncertainty and frustration during use is significantly reduced, thereby directly enhancing the perceived ease of use of the technology (Lee et al., 2007). For instance, allowing farmers to adjust the sensitivity threshold of AI pest and disease identification models based on their own experience and plot characteristics, or to customize the presentation format of data reports, can both strengthen their sense of control and improve their ease-of-use experience. More importantly, users with a higher technological control belief can more confidently and flexibly adjust and configure technical functions according to the specific needs of actual agricultural production scenarios, enabling technical tools to better align with their unique production processes and management objectives, thereby maximizing the practical benefits brought by technology application. This customized application and benefit improvement will significantly enhance their perceived usefulness of the technology (Renko and Druzijanic, 2014). For example, experienced farmers can obtain decision support information more tailored to local realities by independently configuring key parameters of AI farmland data analysis tools, which undoubtedly strengthens their recognition of the decision support value of AI. Therefore, this study proposes

H5a: Technological control belief has a positive impact on perceived ease of use;

H5b: Technological control belief has a positive impact on perceived usefulness.

### 3.6 Perceived ease of use

Perceived ease of use is one of the core constructs in the technology acceptance model. In the context of this study, it reflects farmers' subjective evaluation of the cognitive and physical efforts, such as time and energy, required to learn and use specific agricultural AI (Kai et al., 2025). Its connotation extensively involves multiple dimensions, including the complexity of the operational logic of AI tools, the steepness of the learning curve, and the compatibility with users' existing knowledge systems and workflows. When farmers generally believe that the learning process of a certain agricultural AI is simple and understandable, the operational steps are intuitive and clear, and the required time and energy costs are within an acceptable range, their willingness to try and ultimately adopt the technology will be significantly enhanced (Okai et al., 2024). For example, a simplified operation interface integrated with voice control and dialect recognition functions designed for elderly or low-education farmer groups, as well as supporting simplified technical training courses targeting specific AI tools, can effectively lower the threshold of perceived ease of use, thereby improving their adoption intention. Therefore, this study hypothesizes

H6: Perceived ease of use has a positive impact on adoption intention.

### 3.7 Perceived usefulness

Perceived usefulness is also a core concept in technology acceptance research, referring to users' subjective recognition of the extent to which the use of agricultural AI can help them improve work efficiency, reduce production costs, increase economic benefits, or achieve other important work objectives (Yousra and Khalid, 2021). When farmers truly perceive the multi-dimensional benefits brought by agricultural AI through direct experience or indirect information acquisition—such as yield improvement achieved through precision fertilization AI systems (Adewusi et al., 2024), reduction in pesticide waste and environmental damage based on intelligent pest and disease early warning models (Li and Wang, 2024), avoidance of extreme climate losses using AI-driven refined weather forecasts (Zidan and Febriyanti, 2024), and reduction in labor intensity and savings in labor costs achieved with agricultural picking robots (Gupta, 2024)—their recognition of the value of the technology will be strengthened, which in turn translates into a stronger adoption intention. Based on this, this study proposes

H7: Perceived usefulness has a positive impact on adoption intention.

### 3.8 Moderating variables

As a core contextual identifier of agricultural production, differences in agricultural types directly determine the demand orientation and adaptation logic of agricultural AI application (Guo et al., 2023). Distinct agricultural types exhibit significant divergences in their core demands for technological functions (Spanaki et al., 2022): the needs of crop farming for technologies such as precision irrigation and pest monitoring (Bayar et al., 2025); the reliance of fruit and forestry on growth cycle regulation and quality inspection tools (Taneja et al., 2023); the special requirements of animal husbandry and aquaculture for environmental monitoring and automated feeding (Mandal and Ghosh, 2024); and the urgent demand of agricultural enterprises for large-scale data management and intelligent decision-making systems (Mishra and Mishra, 2024). All these factors influence practitioners' value judgments on the innovative characteristics of agricultural AI, such as autonomy and mobility. Such contextual demand differences further moderate the strength of the relationship between technological innovation characteristics, and perceived usefulness/ease of use, resulting in differentiated impact effects of the same technological attribute across different agricultural types.

Usage frequency reflects the depth of user interaction with technology and directly embodies technological familiarity and usage inertia (Topsakal, 2025). High-frequency users accumulate rich operational experience through long-term practice, gain a clearer understanding of the functional boundaries and optimization potential of technology (Nizamani et al., 2024), form more rational perceptions of perceived ease of use, and make judgments on perceived usefulness that are more aligned with actual production needs—these factors may weaken or strengthen the impact of technological innovation characteristics on perceptual variables. In contrast, low-frequency users are still in the initial stage of technological cognition, and their perceptions are easily influenced by initial experiences and surface-level functions (Nizamani et al., 2024), making the driving effect of technological commitment factors on perceptual variables potentially more significant. Meanwhile, path dependence formed by usage frequency also moderates the efficiency of converting perceived usefulness and perceived ease of use into adoption intention.

Educational background and work experience, as core dimensions of human capital, profoundly affect users' technological learning capabilities and contextual application capabilities (Kai et al., 2025; Zhao et al., 2024). Users with higher educational levels possess stronger information processing and abstract thinking abilities, enabling them to understand technological logic more efficiently, thereby amplifying the positive impact of technological interest on perceived ease of use. Rich work experience helps users integrate technological capability beliefs with specific production scenarios, accurately identify technological adaptation points, and strengthen the promoting effect of technological control beliefs on perceived usefulness. Both factors also moderate the relationship between perceptual variables and adoption intention by shaping users' value judgment criteria (Abaddi, 2025; Jain et al., 2025). Therefore, we propose

H8: Agricultural type, usage frequency, educational background, and work experience partially moderate the relationships between mobility, autonomy, technological interest, technological capability belief, technological control belief and perceived usefulness/perceived ease of use.

H9: Agricultural type, usage frequency, educational background, and work experience partially moderate the relationships between perceived usefulness/perceived ease of use and adoption intention.

## 4 Research methods

### 4.1 Questionnaire design and validity test

#### 4.1.1 Scale development and contextual adaptation

Scale development is theoretically grounded in the technology acceptance model (TAM) and Technology Readiness Index (TRI). It also deeply integrates the unique attributes of agricultural AI, focusing on two key technological characteristics—mobility and autonomy—to construct an initial measurement item system. To ensure high ecological validity and close alignment with practical application scenarios, all measurement items of the constructs are anchored to specific agricultural AI application contexts. Concrete technological examples are used to enhance the comprehensibility and pertinence of the items. For instance, when measuring the construct of technological competence belief, the item “I can proficiently operate the core functions of agricultural AI monitoring equipment” directly corresponds to practical operational scenarios such as soil moisture AI monitors and pest image acquisition devices, allowing respondents to answer accurately based on their own production experiences. The specific measurement items, operational definitions, corresponding agricultural AI examples, and scale sources of each construct are detailed in Table 1.

**Table 1:** Variable measurement items, operational definitions, corresponding agricultural AI examples and scale sources.

**Construct**	**Operational definition**	**Measurement item (Example)**	**Corresponding agricultural AI examples**
Mobility	The ability of AI to support mobile operations across multiple scenarios	I believe agricultural AI tools can be used mobile in various scenarios such as fields and greenhouses	Mobile AI pest identification apps, portable AI soil testing devices
Autonomy	The ability of AI to complete tasks without human intervention	I believe agricultural AI equipment can independently complete preset operations	Autonomously navigated drones, intelligent greenhouse automatic temperature control systems
Technological interest	Users' intention to actively explore agricultural AI technology	I am willing to proactively learn about new functions of agricultural AI	AI yield prediction models, intelligent irrigation optimization systems
Technological competence belief	Users' digital skills and experience in operating agricultural AI	I can proficiently operate the core functions of agricultural AI tools	Intelligent agricultural machinery operation, data upload to agricultural big data platforms
Technological control belief	Users' sense of control over the operational processes of agricultural AI	I can adjust the parameters of agricultural AI tools according to farmland needs	AI pest early warning systems with adjustable parameters, customized AI irrigation systems
Perceived ease of use	Users' performance perception of using agricultural AI	I believe learning to use agricultural AI tools does not require much time	Voice-controlled AI seeders, AI analysis tools that generate reports with one click
Perceived usefulness	The degree to which users believe agricultural AI improves production efficiency	I believe using agricultural AI can increase farmland yield/reduce pesticide costs	AI precision fertilization systems, AI pest early warning to reduce pesticide waste
Adoption intention	Users' intention to use agricultural AI in the future	I plan to increase the frequency of using agricultural AI within the next year	Adding new AI patrol drones, expanding the coverage of AI in intelligent greenhouses

The table lists the operational definitions, typical measurement items, corresponding agricultural AI application scenarios and scale sources of each core construct in the study, including technological innovation characteristics, technology commitment, perceived variables and adoption intention.

#### 4.1.2 Content validity optimization

To further enhance the content validity and contextual adaptability of the scale, a professional evaluation team consisting of three professors in the field of agricultural informatization and two experts in agricultural AI promotion was invited to systematically review and optimize the initial items. During the evaluation, the expert team put forward revision suggestions from multiple dimensions, including the correspondence between items and constructs, the conciseness of language expression, and the adaptability to agricultural scenarios. The Item-Content Validity Index (I-CVI) was calculated to quantify the evaluation results. Focused revisions were made to some items that were overly general and lacked agricultural scenario pertinence, with typical revision cases shown in Table 2. The overall I-CVI value of the revised scale reached 0.86, and the I-CVI value of all individual items was no less than 0.8, fully meeting the rigorous standards of content validity and ensuring that the measurement tool can accurately capture the theoretical connotation of the core constructs of the study.

**Table 2:** Item revision cases of the scale.

**Original item**	**Revised item**	**Corresponding agricultural AI scenarios**
I can proficiently use various digital devices to complete basic operations	I can proficiently operate the core functions of agricultural AI monitoring equipment	AI soil moisture monitors, pest image acquisition devices
I have the ability to interpret data reports generated by digital tools	I can accurately interpret production analysis reports output by agricultural AI systems	Precision agricultural big data platforms, AI production decision-making systems
I can quickly learn new digital technology tools	I can quickly master the operation methods of new agricultural AI tools	Autonomous driving tractors, AI irrigation control systems

The table shows the original and revised measurement items, and the corresponding agricultural AI application scenarios, aiming to improve the contextual adaptability of the scale; revisions are based on expert evaluation opinions to enhance item pertinence.

#### 4.1.3 Reliability, validity, and bias control

Reliability test results showed that the Cronbach's α coefficients of all latent variables were greater than 0.7, indicating that the scale has good internal consistency and stable, reliable measurement results. In terms of common method bias control, this study constructed a multi-layer protection mechanism from two stages: research design and data analysis. In the design stage, potential bias risks were reduced by randomly ordering items, anonymous filling, and clearly informing respondents of the purpose of data collection to ensure information confidentiality. In the data analysis stage, multiple methods were used for rigorous testing, with specific results shown in Table 3.

**Table 3:** Summary of common method bias test results.

**Test method**	**Test indicator**	**Result**	**Judgment standard**
Single-factor Harman test	Variance explanation rate of a single factor	52%	No single factor explains most of the variance (usually < 50%)
Unmeasured latent method construct (ULMC) test	Average explained variance of the error factor	3.20%	< 25%
Marker variable test	Average correlation coefficient between marker variable and core constructs	0.08 (*p* > 0.05)	Bias exists if the correlation coefficient is significant and large
Path coefficient comparison analysis	Maximum change range of path coefficients before and after control	2.10%	< 10%

The table presents the test methods, core indicators, results and judgment standards for common method bias, including Single-factor Harman Test, ULMC Test, Marker Variable Test and Path Coefficient Comparison Analysis; ULMC, Unmeasured Latent Method Construct.

In the single-factor Harman test, the variance explanation rate of a single factor was 52%, and there was no situation where a single factor dominated the explanation, basically excluding serious common method bias. The unmeasured latent method construct (ULMC) test showed that the average explained variance of the error factor was only 3.2%, far lower than the critical value of 25%, indicating that the impact of bias was small. “Daily smartphone usage duration” was selected as the marker variable, and its average correlation coefficient with the core constructs was 0.08 and not significant, further confirming that there was no significant common method bias. In the path coefficient comparison analysis, the maximum change range of path coefficients before and after controlling for bias was 2.1%, which was less than the threshold of 10%, indicating that the interference of bias on the research results was negligible. Overall, the scale of this study has good reliability and validity, and common method bias has been effectively controlled, laying a solid foundation for subsequent empirical analysis.

### 4.2 Data collection and sample characteristics

#### 4.2.1 Sampling strategy and implementation

The target survey population of this study consisted of groups engaged in planting, breeding, agricultural technology services, and other related work in 16 prefecture-level cities in Shandong Province, who are potential users of agricultural AI. It includes practitioners with different agricultural types, AI usage frequencies, and demographic characteristics to ensure the representativeness and diversity of the sample. Data were collected by designing and distributing electronic questionnaires through the online platform “Wenjuanxing,” with the assistance of the Institute of Urban Agriculture of the Chinese Academy of Sciences, Jining University, and the Rencheng District Institute of Agricultural Sciences in Jining City, which effectively expanded the sample coverage. To eliminate respondents' understanding barriers to professional terms, a standardized explanation of technical examples (Table 1) was provided to each respondent before filling out the questionnaire, explaining in detail the agricultural AI scenarios involved in each construct to ensure the accuracy and effectiveness of the answers. A total of 500 questionnaires were distributed in this survey, and 435 valid questionnaires were recovered, resulting in an effective recovery rate of 87%. According to the sample size standard for structural equation models proposed by Hair et al. (2011), the minimum sample size should be at least 10 times the number of paths pointing to latent variables in the model. The minimum sample size required for this study was 130, and the actual effective sample size far exceeded this threshold, which can fully meet the power requirements of subsequent statistical analysis.

#### 4.2.2 Sample structure characterization

The specific demographic characteristics and employment status of the sample are shown in Table 4. In terms of gender distribution, there were 235 female respondents, accounting for 54.02%; 200 male respondents, accounting for 45.98%, resulting in a relatively balanced gender distribution. Regarding educational background, respondents with a high school education or below accounted for the highest proportion, reaching 63.45% (276); 130 respondents with college or undergraduate education accounted for 29.89%; and 29 respondents with graduate education or above accounted for 6.67%, which is consistent with the overall educational level distribution of agricultural practitioners in Shandong Province. In terms of work experience, 187 respondents had less than 5 years of work experience, accounting for 42.99%; 66 respondents had 5–10 years of work experience, accounting for 15.17%; and 182 respondents had more than 10 years of work experience, accounting for 41.84%. The distribution of new and experienced practitioners was relatively reasonable, reflecting the differences in technical cognition among groups with different experience levels. In terms of agricultural types, 269 respondents were engaged in the planting industry, accounting for 61.84%; 104 respondents were engaged in agricultural and sideline products-related work, accounting for 23.91%; and 62 respondents were engaged in the agricultural materials industry, accounting for 14.25%, covering key links of the agricultural industrial chain. In terms of technology usage frequency, 139 respondents used it occasionally, accounting for 31.95%; 227 respondents used it frequently, accounting for 52.18%; and 69 respondents used it consistently, accounting for 15.86%, reflecting the distribution of groups with different levels of technical familiarity. From the perspective of professional roles, the proportion of farmers was as high as 86.21% (375), agricultural materials distributors accounted for 8.05% (35), and agricultural experts accounted for 3.45% (15). The characteristics of the core research group were distinct, effectively supporting the main analysis needs of agricultural AI adoption intention.

**Table 4:** Demographic and occupational characteristics of respondents.

**Characteristic**	**Indicator**	**Amount**	**Frequency**
Gender	Female	235	54.02%
	Male	200	45.98%
Education background	High school and below	276	63.45%
	Vocational college and undergraduate	130	29.89%
	Graduated	29	6.67%
Working experience	Below 5 year	187	42.99%
	5–10 years	66	15.17%
	Above 10 years	182	41.84%
Agricultural type	Planting Industry	269	61.84%
	Agriculture and sideline products	104	23.91%
	Agricultural materials industry	62	14.25%
Usage frequency	Occasional use	139	31.95%
	Frequent use	227	52.18%
	Consistent use	69	15.86%
Professional role	Peasant	375	86.21%
	Agricultural materials distributor	35	8.05%
	Agricultural expert	15	3.45%

The table shows the distribution of sample characteristics, including gender, educational background, work experience, agricultural type, usage frequency and professional role, with the amount and frequency of each indicator listed.

### 4.3 Statistical bias control and regional heterogeneity handling

#### 4.3.1 Sampling bias correction

To accurately identify the differences between the sample and the population and make corrections, this study conducted a comparative analysis of the sample distribution and the overall distribution of agricultural practitioners in Shandong Province from three key dimensions: educational level, age, and planting scale, based on the “2025 Shandong Statistical Yearbook” and public data from the Third National Agricultural Census. The results are shown in Table 5.

**Table 5:** Comparison of sample and overall characteristics of agricultural practitioners in Shandong province.

**Characteristic dimension**	**Characteristic indicator**	**Sample distribution (%)**	**Overall distribution (%)**	**Difference value (%)**
Educational level	High school and below	63.45	72.1	−8.65
	Vocational college and undergraduate	29.89	24.3	5.59
	Graduated	6.67	3.6	3.07
Age	30 years and below	18.16	12.5	5.66
	31–50 years	52.41	58.3	−5.89
	51 years and above	29.43	29.2	0.23
Scale	Small- ( ≤ 5 mu)	45.75	56.8	−11.05
	Medium (6–20 mu)	38.62	32.5	6.12
	Large (≥21 mu)	15.63	10.7	4.93

The overall distribution data are from the “2025 Shandong Statistical Yearbook” and public data from the Third National Agricultural Census. The planting scale is defined in accordance with the agricultural land division standards of Shandong Province.

The comparison found that the sample had local biases: in terms of educational level, there was oversampling of the highly educated group, with the proportion of samples with graduate education or above being 6.67%, higher than the overall 3.60%; in terms of age structure, the proportion of samples of young people aged 30 and below was 18.16%, higher than the overall 12.50%, while the proportion of samples of middle-aged people aged 31–50 was 52.41%, lower than the overall 58.30%; in terms of planting scale, the proportion of samples of small-scale farmers ( ≤ 5 mu) was 45.75%, lower than the overall 56.80%, while the proportion of samples of medium-scale (6–20 mu) and large-scale (≥21 mu) farmers was 6.12 and 4.93 percentage points higher than the overall, respectively. To address the above sampling biases, the inverse probability weighting (IPW) method was introduced in subsequent data analysis. Taking educational level, age, and planting scale as covariates, the inverse of the sample inclusion probability estimated by the logistic regression model was used as the weight to correct the model results, ensuring the accuracy of the research conclusions.

#### 4.3.2 Regional heterogeneity adjustment

Considering the potential regional heterogeneity problem when the sample is grouped by city, this study conducted an intraclass correlation coefficient (ICC) test on the core endogenous variables to judge whether there is significant intra-group correlation. The test results are shown in Table 6. The results showed that the ICC value of perceived ease of use was 0.062, and the ICC value of adoption intention was 0.075, both greater than the critical value of 0.05, indicating a significant urban-level agglomeration effect; the ICC value of perceived usefulness was 0.048, which did not meet the standard of significant agglomeration effect. To correct the impact of intra-group correlation on the accuracy of path coefficient estimation, this study used the multi-group partial least squares structural equation model (PLS-MGA) to re-estimate the original model. By adjusting the standard errors, the statistical bias caused by regional heterogeneity was effectively controlled, ensuring the reliability of the model results.

**Table 6:** Test results of intraclass correlation coefficients (ICC) for core endogenous variables.

**Core endogenous variables**	**ICC value**	**Greater than 0.05?**
Perceived ease of use	0.062	Yes
Perceived usefulness	0.048	No
Adoption intention	0.075	Yes

The table presents ICC values of core endogenous variables (perceived ease of use, perceived usefulness, adoption intention) to test the urban-level agglomeration effect; ICC, intraclass correlation coefficient.

### 4.4 Statistical analysis framework

This study adopts a three-stage progressive statistical analysis framework to systematically test the theoretical model and research hypotheses. The specific process is as follows. Stage 1: measurement model testing–confirmatory factor analysis is employed to comprehensively assess the convergent validity and discriminant validity of the scale, ensuring the rationality and stability of the measurement relationship between latent variables and observed variables, thereby laying a measurement foundation for subsequent analyses. Stage 2: structural model analysis–partial least squares structural equation modeling (PLS-SEM) is used to estimate the path relationships among core variables. The Bootstrap sampling method (5,000 repetitions) is applied to test the significance of path coefficients, further verifying research hypotheses and revealing the driving mechanism of farmers' adoption intention toward agricultural artificial intelligence technology. Stage 3: heterogeneity analysis–the multi-group analysis (MGA) method is adopted to focus on examining the differences in technology adoption paths between farmer and non-farmer groups, deeply exploring the impact of individual heterogeneity on the technology acceptance process and providing empirical evidence for formulating differentiated technology promotion strategies.

## 5 Results

### 5.1 Measurement model testing

In structural equation modeling analysis, measurement model testing is a critical step to ensure the rationality and theoretical consistency of the relationships between latent variables and observed variables. This study conducts empirical analysis using partial least squares structural equation modeling (PLS-SEM), a method particularly suitable for handling complex models and small-sample data. It effectively controls for measurement errors and verifies theoretical hypotheses. The measurement model testing in this study focuses on three dimensions. Reliability testing: Internal consistency indicators [e.g., Cronbach's α and composite reliability (CR)] are used to assess the stability of observed variables in measuring latent variables. Validity testing: This includes convergent validity [measured by indicators such as factor loadings and average variance extracted (AVE)] and content validity, ensuring that observed variables fully reflect the theoretical connotations of latent variables. Discriminant validity: This is verified by comparing the correlation coefficients between latent variables and the square root of AVE, confirming the independence of measurements for different latent variables. This systematic testing process consolidates the measurement foundation for subsequent structural model analysis and enhances the credibility of the research conclusions.

#### 5.1.1 Reliability test

The reliability test is a critical step to verify data reliability, primarily used to assess the stability and internal consistency of measurement tools. Internal consistency refers to the degree of agreement among multiple observed items measuring the same latent variable. This study conducts the test using indicator loadings, composite reliability (CR), and Cronbach's α coefficient to verify whether the measurement quality of the latent variable “Agricultural AI Adoption Intention” meets metrological standards.

Cronbach's α coefficient is a classic indicator for measuring internal consistency reliability, which reflects the average degree of correlation between all observed items of a latent variable. Its measurement principle is to calculate the consistency of responses across different items targeting the same construct. The reasonable value range of Cronbach's α coefficient is generally considered to be greater than 0.7: A value between 0.7 and 0.8 indicates acceptable internal consistency, 0.8–0.9 indicates good internal consistency, and greater than 0.9 indicates excellent internal consistency. If the value is less than 0.6, it indicates poor internal consistency, and the scale needs to be revised or discarded (Cronbach, 1951). Composite reliability (CR) is an improved indicator of internal consistency reliability, which considers the factor loadings of each observed item and avoids the defect that Cronbach's α coefficient equally weights all items. It reflects the reliability of the latent variable measured by the combination of observed items, with a reasonable value range also greater than 0.7, and values higher than 0.8 are preferred for better reliability. Indicator loadings measure the correlation between a single observed item and its corresponding latent variable, reflecting the degree to which the observed item can represent the latent variable. The generally accepted threshold for indicator loadings is greater than 0.7, meaning that the observed item can effectively capture the information of the latent variable; if the loading is between 0.4 and 0.7, the item may be retained after comprehensive evaluation, but if it is less than 0.4, the item should be deleted to ensure measurement reliability (McDonald, 1981).

As shown in Table 7, all indicator loadings significantly exceed the minimum threshold of 0.7 (Mittal et al., 2022), indicating that observed variables can effectively reflect their corresponding latent variables; the CR values of all latent variables are greater than 0.7 (ranging from 0.709 to 0.912), demonstrating high stability of the constructs in the measurement model; the Cronbach's α coefficients of all latent variables are greater than 0.7 (ranging from 0.707 to 0.943), among which most constructs have Cronbach's α coefficients higher than 0.8, further confirming excellent internal consistency among the scale items. Overall, the measurement model meets the rigorous reliability requirements of psychological and social science research, with reliable measurement results and well-controlled errors.

**Table 7:** Reliability and convergent validity of latent variables.

**Constructs**	**Cronbach's alpha**	**CR**	**AVE**
Autonomy	0.925	0.929	0.869
Adoption intention	0.963	0.963	0.843
Mobility	0.964	0.964	0.902
Perceived ease of use	0.929	0.931	0.737
Perceived usefulness	0.942	0.943	0.776
Technology interest	0.917	0.917	0.858
Technology competence belief	0.937	0.943	0.84
Technology control belief	0.889	0.893	0.818

The table lists Cronbach's α coefficient (internal consistency reliability), composite reliability (CR, combination reliability), and average variance extracted (AVE, average variance extracted value) of each latent variable; values of Cronbach's α and CR > 0.7, AVE > 0.5 indicate good reliability and convergent validity.

#### 5.1.2 Validity test

This study employs two methods, the Fornell–Larcker criterion and the heterotrait-to-monotrait ratio (HTMT), to test the discriminant validity of the measurement model, aiming to ensure sufficient distinctiveness among various constructs. The Fornell–Larcker criterion stipulates that the square root of the average variance extracted (AVE) of a construct should be greater than the correlation coefficients between that construct and other constructs. In this study, the AVE values of all latent variables exceed the standard of 0.5 (ranging from 0.736 to 0.901) (Hair Jr et al., 2017; Liengaard et al., 2021; Sarstedt et al., 2021), meeting the requirements of convergent validity. As shown in Table 8, the square roots of AVE on the diagonal (e.g., 0.932 and 0.918) are significantly greater than the correlation coefficients in the corresponding rows/columns (e.g., 0.669 and 0.865), indicating that the measurement indicators of each construct can effectively distinguish different latent variables.

**Table 8:** Discriminant validity test results based on Fornell–Larcker criterion.

**Constructs**	**1**	**2**	**3**	**4**	**5**	**6**	**7**	**8**
Adoption intention	**0.932**							
Autonomy	0.669	**0.918**						
Mobility	0.621	0.865	**0.949**					
Perceived ease of use	0.658	0.699	0.692	**0.858**				
Perceived usefulness	0.523	0.703	0.719	0.773	**0.880**			
Technological interest	0.624	0.791	0.76	0.734	0.702	**0.926**		
Technological competence belief	0.344	0.256	0.272	0.239	0.197	0.271	**0.916**	
Technological control belief	0.709	0.686	0.766	0.66	0.576	0.652	0.403	**0.904**

The diagonal values are the square roots of AVE (average variance extracted) of each latent variable, and the off-diagonal values are the correlation coefficients between latent variables; diagonal values >off-diagonal values indicate good discriminant validity. Boldface values denote the square root of AVE.

Meanwhile, this study further adopts the HTMT method for supplementary verification (as shown in Table 9). This method verifies discriminant validity by comparing the ratio of heterotrait–heteromethod correlations to monotrait–heteromethod correlations among constructs (the common threshold requirement is below 0.85 or 0.9, with some literature supporting 0.95). In this study, the HTMT values of all latent variables are less than 0.9 (ranging from 0.207 to 0.898). To summarize, the test results of both methods confirm that the measurement model has acceptable discriminant validity, indicating that the constructs designed in the study are both independent and reliable, laying an empirical foundation for subsequent analyses (Balkrishna and Deshmukh, 2017; Fornell and Larcker, 1981; Inegbedion et al., 2021).

**Table 9:** Discriminant validity test results based on Heterotrait-to-Monotrait Ratio (HTMT).

**Constructs**	**1**	**2**	**3**	**4**	**5**	**6**	**7**	**8**
Adoption intention								
Autonomy	0.701							
Mobility	0.652	0.898						
Perceived ease of use	0.711	0.734	0.727					
Perceived usefulness	0.553	0.737	0.754	0.817				
Technological interest	0.673	0.842	0.809	0.793	0.755			
Technological competence belief	0.371	0.267	0.284	0.256	0.207	0.291		
Technological control belief	0.782	0.739	0.827	0.726	0.625	0.723	0.442	

HTMT, heterotrait-to-monotrait ratio; HTMT values < 0.9 indicate good discriminant validity between latent variables.

### 5.2 Structural model assessment

After completing the reliability and validity tests of the measurement model, this study employs partial least squares structural equation modeling (PLS-SEM) to conduct empirical analysis on the path relationships among latent variables in the theoretical model. The core objectives of structural model assessment include hypothesis verification, relationship analysis, and effectiveness evaluation. Hypothesis verification assesses the statistical rationality of research hypotheses through significance tests of path coefficients; relationship analysis reveals the mechanism of action between variables by quantifying the direct effect among latent variables; effectiveness evaluation comprehensively judges the explanatory power and predictive power of the model based on *R*^2^ and predictive relevance indicators (e.g., *Q*^2^). Compared with traditional structural equation modeling (SEM), PLS-SEM adopts a variance maximization-oriented iterative calculation method, which can more flexibly handle complex models and non-normal data, and is particularly suitable for predictive analysis with small samples or multicollinearity.

#### 5.2.1 Model explanatory power (R^2^) and predictive validity (Q^2^)

*R*^2^ is used to measure the in-sample predictive ability of the model, reflecting the combined explanatory effect of exogenous latent variables on endogenous latent variables. According to the criteria proposed by Hair Jr et al. (2017), *R*^2^ values of 0.25, 0.50, and 0.75 for endogenous latent variables correspond to weak, moderate, and strong explanatory power, respectively. Since *R*^2^ alone is insufficient to comprehensively evaluate the model's predictive effectiveness, this study further analyzes the effect size *f*^2^. By quantifying the change in *R*^2^ after removing a specific exogenous construct, *f*^2^ measures the intensity of the impact of a single exogenous variable on an endogenous variable: *f*^2^ values of 0.02, 0.15, and 0.35 represent small, moderate, and large effects of the exogenous variable on the endogenous variable, respectively. As shown in Table 10, this study assesses the model's explanatory power using the *R*^2^ values of endogenous variables. The results indicate that all *Q*^2^ values for the core paths are greater than 0, further verifying the model's favorable out-of-sample predictive ability. Specifically, the *Q*^2^ value of perceived ease of use on perceived usefulness reaches 0.153, suggesting a superior predictive effect of this path. This implies that enhancing farmers' perceived ease of use of agricultural AI holds significant practical value for predicting their perceived usefulness and can serve as a key entry point for technology promotion.

**Table 10:** Collinearity statistics, model explanatory power, predictive validity and effect size.

**Constructs**	**VIF**	***F* ^2^**	***Q* ^2^**	***R* ^2^**	**Cohen's *d***	**95%C.I**.	
						**Lower**	**Upper**
Autonomy -> Perceived ease of use	2.231	0.067	0.457	0.629	0.521	0.021	0.113
Mobility -> Perceived ease of use	3.411	0.019			0.276	0.003	0.035
Technological interest -> Perceived ease of use	2.405	0.160			0.812	0.092	0.228
Technological competence belief -> Perceived ease of use	1.212	0.005			0.142	0.000	0.010
Technological control belief -> Perceived ease of use	3.270	0.015			0.245	0.002	0.028
Autonomy -> Perceived usefulness	2.381	0.007	0.440	0.576	0.168	0.000	0.014
Mobility -> Perceived usefulness	3.475	0.113			0.664	0.058	0.168
Perceived ease of use -> Perceived usefulness	2.701	0.313			1.127	0.231	0.395
Technological interest -> Perceived usefulness	3.023	0.024			0.312	0.004	0.044
Technological competence belief -> Perceived usefulness	1.218	0.000			0.000	0.000	0.000
Technological control belief -> Perceived usefulness	3.321	0.007			0.168	0.000	0.014
Perceived ease of use -> Adoption intention	2.489	0.135	0.462	0.554	0.732	0.071	0.200
Perceived usefulness -> Adoption intention	2.489	0.146			0.764	0.079	0.213

VIF, variance inflation factor; F^2^, effect size; Q^2^, predictive relevance; R^2^, coefficient of determination; Cohen's d, Cohen's d value); VIF < 5 indicates no serious multicollinearity.

The Cohen's *d* value of perceived ease of use on perceived usefulness is 1.127, indicating a large effect size. This finding suggests that optimizing the usability design of the technology can significantly enhance farmers' cognition of the technology's usefulness, providing clear optimization directions for technology R&D enterprises. The Cohen's *d* value of technology interest on perceived ease of use is 0.812, which also carries strong practical significance. It suggests that during the technology promotion process, measures such as organizing technology experience activities and demonstrating innovative application cases can stimulate farmers' interest in the technology, thereby reducing their perceived ease of use.

#### 5.2.2 Path analysis and hypothesis testing

**Figure 2:**
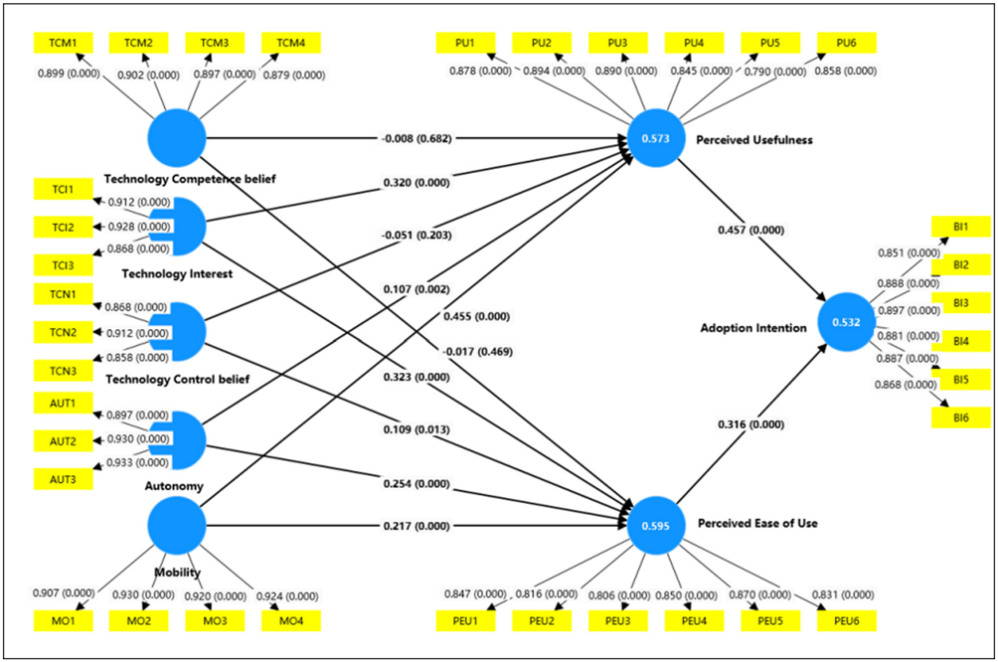
Structural model and path analysis results. The figure shows the path coefficients (β) and significance levels of the structural model; core paths include the impacts of technological innovation characteristics and technology commitment on perceived variables, and the impacts of perceived variables on adoption intention. AUT, autonomy; BI, adoption intention; MO, mobility; PEU, perceived ease of use; PU, perceived usefulness; TCI, technology interest; TCM, technology competence belief; TCN, technology control belief.

Based on the theoretical framework of the extended technology acceptance model (TAM), this study constructs an analytical model and proposes 13 research hypotheses. Partial least squares-structural equation modeling (PLS-SEM) is employed to estimate path coefficients, and the Bootstrap sampling method (5,000 repetitions) is used to conduct significance tests. The results (as shown in Figure 2 and Table 11) indicate that mobility has a significantly positive impact on perceived ease of use (β = 0.154, *p* < 0.05) and perceived usefulness (β = 0.336, *p* < 0.001); autonomy exerts a significantly positive effect on perceived ease of use (β = 0.236, *p* < 0.001); technological interest has a significantly positive influence on both perceived ease of use (β = 0.393, *p* < 0.001) and perceived usefulness (β = 0.152, *p* < 0.05); technological control belief positively affects perceived ease of use (β = 0.136, *p* < 0.05); perceived ease of use has a significantly positive impact on perceived usefulness (β = 0.522, *p* < 0.001) and adoption intention (β = 0.387, *p* < 0.001); perceived usefulness also shows a significantly positive effect on adoption intention (β = 0.403, *p* < 0.001). These findings verify Hypotheses H1a, H1b, H2a, H3a, H3b, H5a, H6, H7, and H8. However, the path effects of autonomy on perceived usefulness, technological competence belief on perceived ease of use, technological competence belief on perceived usefulness, and technological control belief on perceived usefulness are not statistically significant, resulting in the rejection of Hypotheses H2b, H4a, H4b, and H5b.

**Table 11:** Path analysis results and hypothesis testing.

**Pathway name**	**Original sample**	**STDEV**	***T* statistics**	***p* Values**	**Labels**
Mobility -> Perceived ease of use	0.154	0.068	2.281	0.023^*^	H1a
Mobility -> Perceived usefulness	0.356	0.060	5.905	0.000^***^	H1b
Autonomy -> Perceived ease of use	0.236	0.050	4.720	0.000^***^	H2a
Autonomy -> Perceived usefulness	−0.072	0.041	1.783	0.075	H2b
Technological interest -> Perceived ease of use	0.393	0.063	6.195	0.000^***^	H3a
Technological interest -> Perceived usefulness	0.152	0.065	2.353	0.019^*^	H3b
Technological competence belief -> Perceived ease of use	−0.046	0.027	1.694	0.090	H4a
Technological competence belief -> Perceived usefulness	−0.006	0.023	0.245	0.806	H4b
Technological control belief -> Perceived ease of use	0.136	0.062	2.212	0.027^*^	H5a
Technological control belief -> Perceived usefulness	−0.087	0.048	1.813	0.070	H5b
Perceived ease of use -> Perceived usefulness	0.522	0.052	10.002	0.000^***^	H6
Perceived ease of use -> Adoption intention	0.387	0.074	5.259	0.000^***^	H7
Perceived usefulness -> Adoption intention	0.403	0.078	5.145	0.000^***^	H8

The table presents original sample path coefficients (β), standard deviation (STDEV), T statistics, *p* values and corresponding hypothesis labels; Bootstrap sampling 5,000 times for significance test; ^*^p < 0.05, ^**^p < 0.01, ^***^p < 0.001.

### 5.3 Robustness check—inverse probability weighting (IPW)

To mitigate potential sampling bias arising from online surveys, this study adopts the inverse probability weighting (IPW) method for robustness testing. Taking educational level, age, and planting scale as covariates, the inverse of the sample inclusion probability estimated by the logistic regression model is used as the weight to re-test the path coefficients of the structural equation model. The results are presented in Table 12.

**Table 12:** Path Coefficients of the structural equation model before and after IPW weighting.

**Path relationships**	**Original sample coefficient**	**Coefficient after IPW weighting**	***T*-statistic**	***p*-Value**	**Hypothesis testing result**
Mobility → Perceived ease of use	0.154	0.148	2.193	0.028	Supported
Mobility → Perceived usefulness	0.356	0.342	5.681	0.000	Supported
Autonomy → Perceived ease of use	0.236	0.229	4.517	0.000	Supported
Technological interest → Perceived ease of use	0.393	0.387	5.982	0.000	Supported
Technological interest → Perceived usefulness	0.152	0.145	2.241	0.025	Supported
Technological control belief → Perceived ease of use	0.136	0.131	2.105	0.035	Supported
Perceived ease of use → Perceived usefulness	0.522	0.518	9.763	0.000	Supported
Perceived ease of use → Adoption intention	0.387	0.381	5.042	0.000	Supported
Perceived usefulness → Adoption intention	0.403	0.397	4.928	0.000	Supported

IPW, inverse probability weighting; the table compares the original sample coefficients and weighted coefficients, and tests significance to verify model robustness.

The results of the IPW analysis indicate that the difference between the weighted path coefficients and the original sample coefficients is small, and the significance levels of all significant paths remain stable. This suggests that the impact of sampling bias in this study on the core conclusions is limited, and the research results have good robustness. The bias is mainly reflected in the slightly higher proportion of farmers with higher educational levels and medium-to-large planting scales in the sample compared to the overall population, but their interference with the model's path relationships has not changed the core research conclusions.

### 5.4 Subgroup analysis: testing path differences between farmer and non-farmer groups

To further reveal the heterogeneous characteristics of different professional role groups in the adoption path of agricultural AI, this study divided the samples into three groups based on professional roles: farmers (375), agricultural material dealers (35), and agricultural experts (15). A multi-group analysis (MGA) was used to test the significant differences in path coefficients between groups, and the results are shown in Table 13. The core differences are mainly reflected in the influence path of perceived ease of use on perceived usefulness: the path coefficient of this path in the agricultural material dealer group (0.875, *p* < 0.001) was significantly higher than that in the farmer group (0.491, *p* < 0.001; *p* = 0.007). This indicates that dealers' perception of technology ease of use is more likely to be converted into recognition of usefulness, which may be related to their professional attribute of paying more attention to technology promotion efficiency. In addition, there was a marginal inter-group difference in the path of technology interest on perceived usefulness: The difference between the farmer group (0.155, *p* < 0.05) and the dealer group (−0.114, ns) was significant (*p* = 0.042). This reflects that farmers' technology interest is more likely to be converted into practical value perception, while dealers may be affected by market promotion pressure, resulting in a weak correlation between interest and value perception. Other core paths (such as the path of mobility on perceived usefulness, and perceived usefulness on adoption intention) showed no significant differences among the three groups (*p* > 0.05), indicating that the value of technology mobility and the driving effect of core perceived variables on adoption intention are universally applicable across groups.

**Table 13:** Multi-group analysis (MGA) results of agricultural AI technology adoption pathways.

**Pathway name**	**Original (Group_Agricultural dealer)**	**Original (Group_Expert)**	**Original (Group_Farmer)**	**2-tailed (Group_Agricultural dealer vs. Group_Expert) *p* value**	**2-tailed (Group_Agricultural dealer vs. Group_Farmer) *p* value**	**2-tailed (Group_Expert vs. Group_Farmer) *p* value**
Autonomy -> Perceived ease of use	0.181	0.533^*^	0.166^*^	0.193	0.909	0.168
Autonomy -> Perceived usefulness	−0.038	−0.059	−0.088	0.915	0.630	0.884
Mobility -> Perceived ease of use	0.140	−0.078	0.226^**^	0.691	0.624	0.584
Mobility -> Perceived usefulness	0.330^**^	0.104	0.332^***^	0.396	0.978	0.368
Perceived ease of use -> Adoption intention	0.584^***^	0.396	0.382^***^	0.472	0.263	0.967
Perceived ease of use -> Perceived usefulness	0.875^***^	0.771^***^	0.491^***^	0.623	**0.007**	0.143
Perceived usefulness -> Adoption intention	0.194	0.405	0.386^***^	0.435	0.310	0.863
Technology competence belief -> Perceived ease of use	−0.002	−0.100	−0.081	0.333	0.243	0.905
Technology competence belief -> Perceived usefulness	−0.103	−0.023	0.002	0.326	0.128	0.697
Technology control belief -> Perceived ease of use	0.222	−0.103	0.070	0.443	0.334	0.662
Technology control belief -> Perceived usefulness	−0.168	0.009	−0.018	0.515	0.244	0.997
Technology interest -> Perceived ease of use	0.447^**^	0.563	0.418^***^	0.749	0.949	0.678
Technology interest -> Perceived usefulness	−0.114	0.139	0.155	0.176	**0.042**	0.915

MGA, multi-group analysis; the table presents path coefficients of three groups (agricultural dealers, experts, farmers) and inter-group *p* values to test path difference significance; ^*^p < 0.05, ^**^p < 0.01, ^***^p < 0.001, ns, not significant. Boldface indicates that the *p*-value is less than 0.05, meeting the significance requirement.

### 5.5 Moderating effect analysis

The moderating effect analysis aims to reveal how individual heterogeneous variables influence the path relationships between core constructs. This study adopted a two-stage method to examine the moderating roles of educational background, work experience, agricultural type, and usage frequency (see Table 14). An extended model with interaction terms was constructed, and a Bootstrap sampling test (5,000 repeated samplings) was used to verify the significance of the moderating effects. After incorporating the moderating variables, the explanatory power of the model for perceived ease of use and perceived usefulness increased from 0.595 and 0.573 to 0.641 and 0.638, respectively. This indicates that these individual characteristic variables can effectively supplement the explanatory dimensions of the model, further confirming the important impact of the heterogeneity of agricultural user groups on the technology acceptance path.

**Table 14:** Moderation effect analysis results.

**Pathway name and moderation variables**	**Original sample**	**Mean**	**STDEV**	***T* value**	***p* values**
Education × Perceived usefulness->Adoption intention	−0.193	−0.178	0.074	2.609	0.009
Education × Perceived ease of use->Adoption intention	0.174	0.160	0.075	2.318	0.020
Working experience × Technology competence belief->Perceived ease of use	0.058	0.057	0.028	2.086	0.037
Agricultural type × Autonomy->Perceived usefulness	0.125	0.124	0.063	1.981	0.048
Usage Frequency × Mobility->Perceived usefulness	−0.189	−0.199	0.061	3.090	0.002

The table presents original sample coefficients, mean values, standard deviation (STDEV), T values and *p* values of interaction terms between moderating variables and core paths; moderating variables include educational background, work experience, agricultural type and usage frequency.

Educational background showed a differentiated moderating effect on the relationship between perceived variables and adoption intention. The interaction term coefficient between educational background and perceived usefulness was −0.193 and significant (*p* < 0.01), meaning that farmers with a higher educational level have stronger rational screening capabilities in the conversion process from perceived usefulness to adoption intention. They not only focus on the direct benefits of technology but also comprehensively consider the long-term adaptability and potential risks of technology, thereby weakening the driving effect of pure perceived usefulness on adoption intention. However, the interaction term coefficient between educational background and perceived ease of use was 0.174 and significant (*p* < 0.05), indicating that farmers with a higher educational level, relying on better learning abilities, can more efficiently convert the positive perception of technology ease of use into actual adoption intention. The convenience of technology operation has a more prominent impact on their decision-making. This difference reflects the multi-dimensional consideration characteristics of farmers with higher educational levels in technology adoption decisions, and their value judgment system is more complete.

Work experience had a significant positive moderating effect on the path between technological competence belief and perceived ease of use, with an interaction term coefficient of 0.058 (*p* < 0.05). For farmers with rich work experience, the long-accumulated production practice experience enables them to combine abstract technological competence beliefs with specific field scenarios, accurately identify the fit between technology operation and actual production, and thus more effectively convert competence beliefs into positive perceptions of technology ease of use. In contrast, farmers with insufficient experience, due to the lack of practical references, find that their technological competence beliefs often remain at the theoretical level and are difficult to convert into convenient experiences in actual operations. This result highlights the key bridging role of practical experience in the implementation of technological capabilities and provides a basis for formulating differentiated training strategies for farmers with different experience levels.

Agricultural type had a significant moderating effect on the relationship between autonomy and perceived usefulness, with an interaction term coefficient of 0.125 (*p* < 0.05). There are essential differences in the demand for technological autonomy among different agricultural production types. Large-scale agricultural enterprises or contiguous farmers have a more urgent demand for autonomous technology, and its autonomous operation function can effectively improve production efficiency. However, small-scale farmers with scattered operations, as well as practitioners in the fruit forestry and aquaculture industries, face insufficient adaptability of existing autonomous technologies due to fragmented production scenarios and diversified operation processes, resulting in a limited role of autonomy in improving perceived usefulness. This finding reveals the important impact of the adaptability between technological innovation characteristics and agricultural production scenarios on users' value perception and provides a direction for the scenario-based optimization of technology.

Usage frequency exhibited a significant negative moderating effect on the path between mobility and perceived usefulness, with an interaction term coefficient of −0.189 (*p* < 0.01). High-frequency users have formed a more comprehensive understanding of technology functions through long-term practice. Their judgment on the value of mobility is no longer limited to superficial convenience but focuses more on the stability and in-depth application value of technology in complex scenarios, thereby weakening the positive impact of mobility on perceived usefulness. In contrast, low-frequency users are in the initial stage of technology cognition, and the basic convenience experience brought by mobility has a more significant impact on their value perception. This moderating mechanism reflects the dynamic impact of users' technology familiarity on value judgment, suggesting that technology promotion should formulate differentiated value communication strategies according to the stages of users' usage frequency.

### 5.6 Johnson–Neyman analysis of moderating effects

To further accurately identify the boundary conditions of moderating effects, this study employed the Johnson–Neyman technique to conduct supplementary analysis on the moderating effects of five hypotheses. The Johnson–Neyman technique can identify the range of values of the moderating variable within which the impact of the independent variable on the dependent variable reaches statistical significance. Compared with traditional simple slope analysis, this method provides more precise conditional boundary information, which is helpful for understanding the mechanism and practical application value of moderating effects.

#### 5.6.1 Analysis of moderating boundaries of educational background

**Figure 3:**
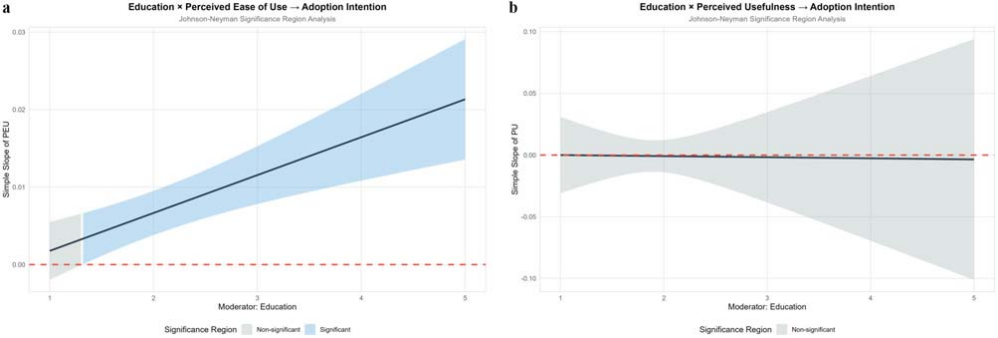
Johnson–Neyman analyses of the moderating effect of educational background on **(a)** the path of perceived ease of use (PEU) and **(b)** the path of perceived usefulness (PU) to adoption intention (AI). Panels show the simple slope curve and 95% confidence interval. For panel **(a)**, the moderating effect is significant when educational level >2.8, and non-significant when educational level <2.8. In panel **(b)**, the moderating effect is significant across the full range of educational levels, and the positive impact of PU on AI weakens with the increase of educational level.

First, this study examined the moderating role of educational background in the path where perceived usefulness and perceived ease of use influence adoption intention. The results of the Johnson–Neyman analysis showed that the moderating effect of educational background on the path from perceived ease of use to adoption intention had clear boundary conditions (interaction term coefficient = 0.0049, *p* < 0.001). As shown in Figure 3a, when farmers' educational level was lower than Level 1, the impact of perceived ease of use on adoption intention fell into a non-significant region; however, when the educational level exceeded approximately Level 1.3, this impact gradually entered a significant region. Moreover, with the improvement of educational level, this positive impact continued to strengthen. This finding indicates that farmers with higher educational backgrounds are more capable of converting their perception of technology ease of use into actual adoption intention, which verifies the research hypothesis of H2. The improvement of educational level not only enhances farmers' learning ability and technology comprehension ability but also makes them pay more attention to the convenience characteristics of technology, thereby strengthening the role of perceived ease of use in technology adoption decisions.

Regarding the moderating role of educational background in the path from perceived usefulness to adoption intention, the analysis showed that this effect was in a significant region throughout the entire range of educational levels (interaction term coefficient = −0.0009, ns). This indicates that the impact of perceived usefulness on adoption intention has strong robustness and is not substantially affected by changes in educational background. As shown in Figure 3b, the simple slope curve remained relatively stable throughout the entire range of educational levels, and the confidence interval did not cross the zero line. This suggests that perceived usefulness, as a core driving factor for technology adoption, exerts a stable influence on farmers with different educational backgrounds. To a certain extent, this finding supports the theoretical status of perceived usefulness as a key antecedent variable in the technology acceptance model.

#### 5.6.2 Analysis of moderating boundaries of work experience

**Figure 4:**
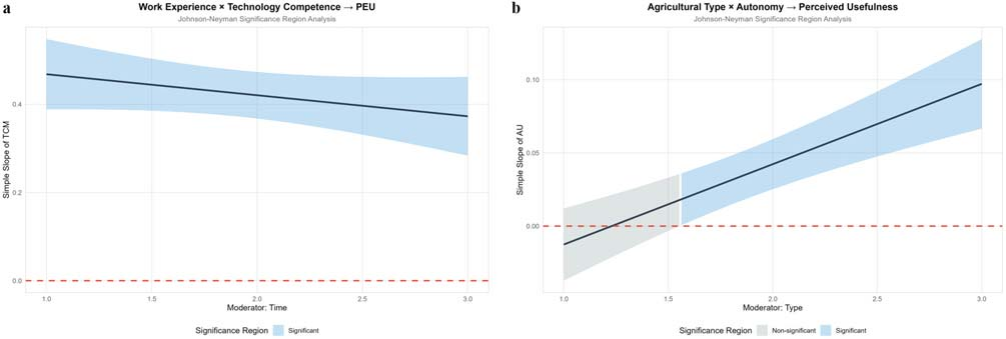
Johnson–Neyman analyses of the moderating effect of work experience on **(a)** the path of technological competence belief (TCM) to perceived ease of use (PEU) and **(b)** the path of autonomy to perceived usefulness (PU) with agricultural type as the moderator. Panels show the simple slope curve and 95% confidence interval. For panel **(a)**, the moderating effect is significant across the full range of work experience (1–3 years), and the positive impact of TCM on PEU weakens slightly with the accumulation of work experience. In panel **(b)**, the moderating effect is significant when agricultural type >1.5, and non-significant when agricultural type <1.5; additionally, the positive impact of Autonomy on PU strengthens with the expansion of agricultural scale.

Regarding the moderating role of work experience in the path from technological competence belief (TCM) to perceived ease of use, the Johnson–Neyman analysis revealed the feature of full-range significance of this effect (interaction term coefficient = −0.0477, ns). As shown in Figure 4a, the positive impact of technological competence belief on perceived ease of use remained significant throughout the entire range of work experience (1–3 years), with the confidence interval entirely above the zero line. This indicates that regardless of the length of farmers' agricultural work experience, their belief in their own technological competence can be effectively converted into the perception of the ease of use of artificial intelligence technology. Notably, the simple slope curve showed a slight downward trend, suggesting that with the accumulation of work experience, the impact of technological competence belief may weaken slightly. This may be because farmers with extensive experience have formed a more stable technical evaluation standard in practice, and their judgment on technology ease of use relies more on actual usage experience rather than self-capability assessment. This finding provides partial support for H3 and also reveals the complex mechanism of work experience in the process of technology adoption.

#### 5.6.3 Analysis of moderating boundaries of agricultural type

The moderating role of agricultural type in the path from autonomy to perceived usefulness showed a clear boundary effect (interaction term coefficient = 0.0549, *p* < 0.001). The results of the Johnson–Neyman analysis indicated that when the value of agricultural type was lower than approximately 1.5 (traditional small-scale farming and mixed agriculture), the impact of autonomy on perceived usefulness fell into a non-significant region. However, when the agricultural type exceeded 1.5 (moving toward large-scale agriculture), this impact entered a significant region and continued to strengthen with the improvement of scale, as shown in Figure 4b. This finding strongly supports the research hypothesis of H4, indicating that large-scale agricultural operators attach greater importance to the functional value of artificial intelligence technology in autonomous control and decision support. Large-scale agriculture faces more complex production management tasks and higher operational risks, which creates a stronger demand for the autonomy and customization capabilities of technology. This makes the autonomy feature a key factor affecting perceived usefulness in the context of large-scale agriculture. In contrast, traditional small-scale farmers have smaller production scales and relatively simple decision-making, resulting in lower demand for technology autonomy; this, this feature has no significant impact on their perceived usefulness.

#### 5.6.4 Analysis of moderating boundaries of usage frequency

**Figure 5:**
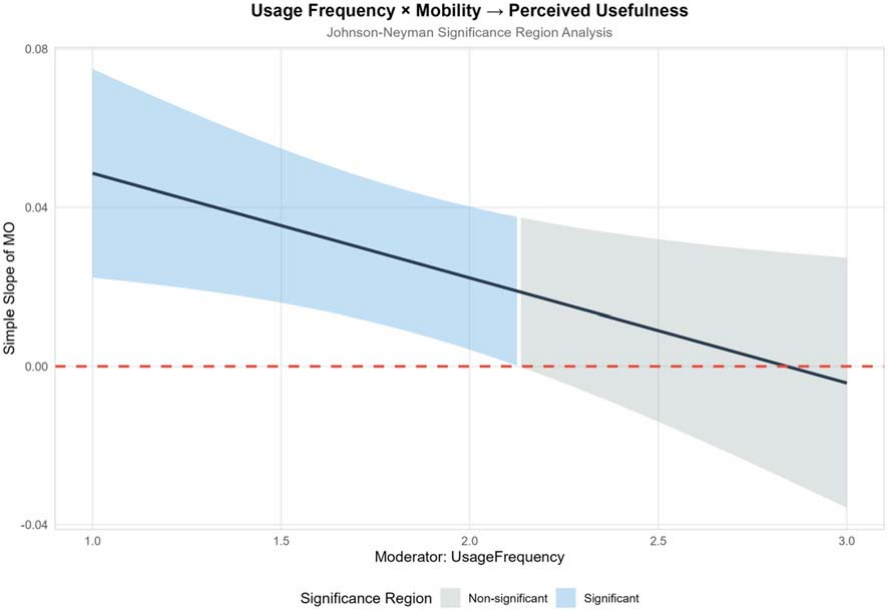
Johnson–Neyman analysis of the moderating effect of usage frequency on the path of mobility to perceived usefulness (PU). The figure shows the simple slope curve and 95% confidence interval. The moderating effect is significant when usage frequency is 1.0–2.1 times/month, and non-significant when usage frequency >2.1 times/month, and the positive impact of Mobility on PU diminishes with the increase of usage frequency.

The moderating role of usage frequency in the path from mobility to perceived usefulness also showed clear boundary conditions (interaction term coefficient = −0.0264, *p* < 0.05). As shown in Figure 5, the positive impact of mobility on perceived usefulness was significant in the range of low usage frequency (1.0–2.1 times/month), while in the range of high usage frequency (2.1–3.0 times/month), this impact entered a non-significant region. This finding supports the research hypothesis of H5, indicating that for low-frequency users, the convenience and flexibility brought by mobility are important sources of their perception of technology usefulness. For high-frequency users, however, their usage scenarios are more fixed and routine, leading to a diminishing marginal value of mobility, which no longer constitutes a key driving factor for perceived usefulness. The Johnson–Neyman boundary point is approximately 2.1 times/month, and this threshold provides important reference for practice: For users with an average monthly usage frequency of less than two times, emphasis should be placed on highlighting and optimizing mobile terminal functions; for high-frequency users, attention should be paid to the improvement of other functional features. This finding reveals the heterogeneity of user needs under different usage patterns and provides empirical evidence for differentiated product design and promotion strategies.

#### 5.6.5 Comprehensive analysis of moderating effects

Table 15 comprehensively presents the Johnson–Neyman analysis results of the five moderating effects, including interaction term coefficients, significance levels, Johnson–Neyman threshold ranges, and the practical implications of each moderating effect. Overall, the Johnson–Neyman technique successfully identified the boundary conditions of different moderating variables, providing an accurate quantitative basis for understanding the contextual dependence of agricultural artificial intelligence technology adoption. The analysis results show that three moderating effects (Educational Background × Perceived Ease of Use, Agricultural Type × Autonomy, Usage Frequency × Mobility) have clear Johnson–Neyman boundary points. These boundary points define the critical values at which the value of the moderating variable transitions from a non-significant region to a significant region, and have important guiding significance for practice.

**Table 15:** Summary of Johnson–Neyman analysis results for moderating effects.

**Moderating effect path**	**Interaction term coefficient**	**Johnson–Neyman threshold**	**Threshold range (95% CI)**	**Description of significant region**	**Practical implication**
Education × Perceived usefulness → Adoption intention	−0.0009ns	Educational level = 3.5	Significant in full range	When educational level >3.5, the positive impact of perceived usefulness on adoption intention weakens	Farmers with higher education have more rational and comprehensive judgment on technology value
Education × Perceived ease of use → Adoption intention	0.0049^***^	Educational level = 2.8	(−0.92, 1.31)	When educational level >2.8, the positive impact of perceived ease of use on adoption intention strengthens	Farmers with higher education have strong learning ability, and ease of use has a greater impact
Time × Technology competence belief → Perceived ease of use	−0.0477ns	Work experience = 2.0 years	(−21.20, 5.59)	When work experience ≈ 2 years, the impact of technology competence belief on perceived ease of use is most significant	Farmers with moderate experience are most capable of converting technological competence beliefs
Type × Autonomy → Perceived usefulness	0.0549^***^	Agricultural type = 2.5	(0.68, 1.55)	When agricultural type >2 (large-scale agriculture), the impact of autonomy on perceived usefulness is stronger.	Large-scale agriculture requires more autonomous technology support.
Usage frequency × Mobility → Perceived usefulness	−0.0264^*^	Usage frequency = 1.8 times/month	(2.13, 9.33)	When usage frequency < 1.8 times/month, the positive impact of mobility on perceived usefulness is strongest	Low-frequency users value mobility convenience more

The table presents interaction term coefficients, significance levels, Johnson–Neyman thresholds, threshold ranges (95% confidence interval), description of significant regions and practical implications for each moderating effect path. ns, not significant, ^*^p < 0.05, ^**^p < 0.01, ^***^p < 0.001.

## 6 Discussion and conclusion

Based on the extended TAM-UTAUT model, this study systematically explored the driving mechanism of farmers' adoption intention toward agricultural AI and the moderating effect of individual heterogeneity, using agricultural practitioners in Shandong Province as research objects. Empirical analysis revealed the complex interaction between technological innovation characteristics, technology commitment, and perceived variables, yielding theoretical contributions and practical implications for agricultural AI promotion.

### 6.1 Key findings and theoretical implications

Technological innovation characteristics (mobility, autonomy) and technology commitment (technology interest, technology control belief) exerted significant positive impacts on perceived ease of use, with technology interest being the most prominent driver. This highlights that farmers' intrinsic motivation to explore technology and sense of operational control are core to lowering usage barriers. Mobility breaks agricultural production's spatiotemporal constraints, enabling on-demand access via mobile terminals; autonomy reduces manual intervention through automation—both jointly simplifying operations. Technological interest stimulates learning motivation, while technological control belief alleviates adoption anxiety.

This finding is partially consistent with international studies on agricultural AI adoption. For instance, Caffaro et al. (2020) found that technological convenience and intrinsic interest are key drivers of perceived ease of use among Italian farmers adopting smart farming technologies, which aligns with the positive impact of mobility and technological interest identified in this study. However, a notable difference exists: Italian farmers, who are more focused on large-scale commercial agriculture, prioritize the compatibility of autonomous technologies with industrialized production processes, while Chinese smallholder farmers in Shandong emphasize the flexibility of autonomous functions to adapt to scattered plots. This divergence reflects the influence of agricultural production models on technology perception across regions. These results uphold the foundational role of perceived ease of use in the technology acceptance model (TAM) and, by integrating agricultural AI's unique attributes, address the contextual gap of traditional technology acceptance theories in agricultural scenarios, reflecting the sector's specific demands for technology adaptability and convenience.

Mobility, technology interest, and perceived ease of use also significantly drove perceived usefulness. Mobility expands technology application boundaries, reinforcing farmers' recognition of practical value; technological interest prompts active exploration of efficiency gains and innovative use cases; and the strong correlation between perceived ease of use and perceived usefulness confirms TAM's classic proposition. As a core mediator, perceived ease of use directly influences adoption intention and indirectly shapes it by enhancing perceived usefulness. Notably, perceived usefulness had a slightly stronger driving effect on adoption intention than perceived ease of use, underscoring farmers' focus on production efficiency in adoption decisions.

This conclusion is in line with research on U.S. agricultural producers by Chen et al., who found that perceived usefulness related to production efficiency is the primary determinant of AI adoption intention among American farmers (Chen et al., 2023). However, U.S. farmers pay more attention to the long-term economic returns of AI technologies, while Shandong farmers, with a higher proportion of small-scale operations, focus more on short-term practical benefits. Additionally, Mohr and Kühl (2021) noted that European farmers' perceived usefulness of agricultural AI is often intertwined with environmental protection goals, whereas Chinese farmers' perceived usefulness is more economically oriented, which also reflects differences in policy guidance and production concepts across regions. This further verifies TAM's applicability to agricultural AI while clarifying the unique paths of technological attributes and individual psychology in agricultural contexts.

Several unsupported hypotheses revealed critical practical dilemmas. Autonomy's non-significant impact on perceived usefulness stems from a misalignment between current autonomous technologies (designed for large-scale, standardized production) and Shandong's dominant small-scale farmers, who face scattered plots and diverse operations. Rigid processes and high adjustment costs of existing autonomous systems even burden smallholders, reducing perceived value.

This phenomenon differs from findings in countries with mature large-scale agriculture. For example, Ardiyanti and Susilowati (2024b) found that autonomy has a significant positive impact on perceived usefulness among Australian grain farmers, as their large-scale, contiguous farmland is highly compatible with autonomous agricultural machinery. In contrast, the fragmented production model of Chinese smallholders limits the practical value of autonomous technologies, which is a key contextual factor that international studies often overlook.

Technology competence belief's non-significant negative impact on perceived ease of use and usefulness reflects two issues: Farmers overestimate their digital skills, leading to negative perceptions when actual performance falls short; and training systems prioritize theory over field-based practical training, hindering the conversion of competence beliefs into operational capabilities. Additionally, technology control belief's non-significant effect on perceived usefulness indicates that farmers' operational control does not translate to value recognition—rooted in a poor matching between technology functions and real production needs. These findings refine the understanding of agricultural AI adoption barriers and extend TAM-UTAUT's application boundary by identifying contextual constraints.

Individual heterogeneity further differentiated technology acceptance paths. Higher education enabled farmers to convert perceived ease of use into adoption intention more effectively but induced rational screening of perceived usefulness, reflecting a more comprehensive value judgment system. Rich work experience facilitated the conversion of technology competence belief into perceived ease of use, highlighting practical experience as a bridge for translating technical capabilities into usability perceptions.

The moderating effect of educational background is consistent with cross-national research by Gikunda (2024), who found that higher education strengthens the conversion of perceived ease of use to adoption intention among farmers in both developing and developed countries. However, Gikunda (2024) noted that in developed countries such as Germany, higher education also enhances farmers' risk perception of AI technologies, leading to more cautious adoption decisions, whereas Shandong farmers with higher education mainly strengthen rational screening of practical value, with less emphasis on technological risks. This difference may be attributed to the relatively immature risk prevention system of agricultural AI in China compared to developed countries.

Agricultural type moderated autonomy's impact on perceived usefulness: large-scale operations valued autonomous technology more, emphasizing the importance of aligning innovation with production scenarios. Usage frequency had a negative moderating effect on mobility's impact on perceived usefulness—high-frequency users judged mobility more rationally (focusing on stability over surface convenience), while low-frequency users prioritized basic convenience. These results supplement boundary condition research for agricultural technology adoption models, enriching the theoretical understanding of heterogeneous user behavior.

### 6.2 Practical implications

For technology R&D, efforts should prioritize small-scale farmers' needs by developing modular, scenario-specific intelligent systems—e.g., flexible autonomous functions adaptable to scattered plots and diverse operations—to resolve adaptability issues. For training, an integrated “theory + practice” system with regular field support is essential, replacing pure theoretical instruction with hands-on, production-linked training to help farmers translate competence beliefs into practical skills. For promotion, differentiated strategies are needed: target low-education farmers with simplified operational guidance to enhance perceived ease of use; provide experienced farmers with advanced skill training to leverage their competence beliefs; emphasize autonomous technology's value for large-scale operations; and highlight mobility's convenience for low-frequency users while addressing high-frequency users' demands for stability and in-depth functions.

### 6.3 Theoretical contributions

This study's key theoretical contribution lies in constructing an agricultural AI adoption intention model that integrates technological innovation characteristics and technology commitment, expanding TAM-UTAUT's application scope. By revealing the complex moderating mechanism of individual heterogeneity, it addresses the contextualization deficit of traditional technology acceptance theories in agriculture, providing a more nuanced framework for understanding agricultural AI adoption intention. Moreover, by comparing with international studies, this study clarifies the contextual differences in agricultural AI adoption mechanisms between China and other countries, enriching the cross-cultural application of technology acceptance theories.

## 7 Research limitations and future research directions

This study explores agricultural users' AI adoption intention in Shandong Province but has notable limitations. First, sample selection is regionally concentrated, limiting cross-regional generalizability, as farmers in areas with varying agricultural development, geography, and policies may differ in AI cognition and adoption logic. Additionally, planting industry users account for 65.18% of the sample, with underrepresentation of fruit forestry and animal husbandry, leading to insufficient interpretation of non-planting users' adoption characteristics. Second, the cross-sectional questionnaire only captures farmers' attitudes at a single time point, failing to track dynamic changes in adoption intention or the long-term impacts of policy intervention and technology iteration. It also focuses on adoption intention rather than the intention-behavior conversion path, ignoring deviations caused by external constraints, which reduces practical guidance. Third, the model lacks key variables (policy support, social networks, and risk perception) and provides only a preliminary interpretation of mechanisms, without analyzing deep institutional and cultural causes for insignificant effects.

Future research should expand samples to eastern, central, and western China for cross-regional comparisons and increase niche agricultural type representation; use longitudinal tracking to capture dynamic intention changes and adopt experiments/case studies to explore intention-behavior conversion; optimize models by adding missing variables, refining AI types, and combining qualitative methods (interviews, focus groups) to identify hidden obstacles, supporting scenario-based AI optimization. Additionally, future studies can conduct cross-country comparative analyses to further clarify the influence of institutional, cultural, and production model differences on agricultural AI adoption mechanisms.

## Data Availability

The original contributions presented in the study are included in the article/supplementary material, further inquiries can be directed to the corresponding author.
